# Adverse Effects of Heat Stress on the Intestinal Integrity and Function of Pigs and the Mitigation Capacity of Dietary Antioxidants: A Review

**DOI:** 10.3390/ani11041135

**Published:** 2021-04-15

**Authors:** Arth David Sol Valmoria Ortega, Csaba Szabó

**Affiliations:** 1 Department of Animal Nutrition and Physiology, Faculty of Agriculture and Food Sciences and Environmental Management, University of Debrecen, Böszörményi Street 138, 4032 Debrecen, Hungary; ortega.david@agr.unideb.hu; 2 Doctoral School of Animal Science, Faculty of Agriculture and Food Sciences and Environmental Management, University of Debrecen, 4032 Debrecen, Hungary

**Keywords:** heat stress, oxidative stress, antioxidants, intestinal integrity and function, pigs, reactive oxygen species

## Abstract

**Simple Summary:**

Heat stress is a significant threat to the pigs’ production performance as it greatly affects various body systems, particularly those that are responsible for nutrient digestion and absorption. Heat-stress-induced stressors such as oxidative stress threaten the integrity and functionality of the intestine by negatively affecting its morphology and histology through reduction of villus height, crypt depth, villus height to crypt depth ratio, mucosal surface and villi sloughing. Its protective function is also compromised as heat stress negatively influences the expression of tight junction proteins and disrupts the tight junction barrier function, leading to endotoxemia. These adverse effects of heat stress can be highly mitigated by supplementing dietary antioxidants, as these substances positively influence the intestinal integrity and function of pigs through the improvement of intestinal morphology and histology. Reduction of blood endotoxin through improved tight junction barrier function and depletion of oxidative stress with enhanced mucosal antioxidant capacity is also evident upon such supplementation.

**Abstract:**

Heat stress (HS) significantly affects the performance of pigs by its induced stressors such as inflammation, hypoxia and oxidative stress (OS), which mightily strain the intestinal integrity and function of pigs. As heat stress progresses, several mechanisms in the intestinal epithelium involved in the absorption of nutrients and its protective functions are altered. Changes in these mechanisms are mainly driven by cellular oxidative stress, which promotes disruption of intestinal homeostasis, leading to intestinal permeability, emphasizing intestinal histology and morphology with little possibility of recovering even after exposure to HS. Identification and understanding of these altered mechanisms are crucial for providing appropriate intervention strategies. Therefore, it is this papers’ objective to review the important components for intestinal integrity that are negatively affected by HS and its induced stressors. With due consideration to the amelioration of such effects through nutritional intervention, this work will also look into the capability of dietary antioxidants in mitigating such adverse effects and maintaining the intestine’s integrity and function upon the pigs’ exposure to high environmental temperature.

## 1. Introduction

Animal welfare and productivity are compromised when livestock animals are exposed to an ambient temperature above their thermoneutral zone [1]. Among the various livestock species, pigs are more sensitive to ambient temperature changes and are susceptible to heat stress (HS). Several types of stressors including inflammation, hypoxia and oxidative stress (OS) are induced by HS in the skeletal muscles and intestine, which adversely affects the pigs’ performance [2,3,4,5]. OS occurs due to an imbalance between the production of reactive oxygen species (ROS) and the available antioxidant defense against them. Various stressors can cause ROS overproduction, and one of them is HS [6,7]. This can then lead to damages to cellular constituents [8]. Physiologically, the organ that is highly sensitive to HS and its induced stressors is the gastrointestinal tract (GIT) [9], as it is the first significant organ being affected [10]. Changes in the pigs’ physiology, metabolism and behavior are evident upon exposure to HS. As an immediate physiological response, pigs tend to increase their peripheral blood flow to promote heat loss; consequently, blood flow to the internal organs, notably the GIT, is reduced [11]. This reduction of blood flow leads to the decrease in the supply of oxygen (hypoxia) and nutrients in the GIT [12], compromising intestinal barrier integrity and function [13]. The intestinal barrier is composed of a single layer of enterocytes and intercellular tight junctions (TJs) and serves as a selective permeable membrane that is responsible for digestion and absorption of nutrients and constitutes the first line of defense against various harmful substances that can enter the intestinal mucosa and systemic circulations [14,15]. HS disrupts TJs in the pigs’ intestine, increasing the circulation of endotoxin [16]. As a result, increased intestinal permeability can occur, leading to a high risk of endotoxemia [17]. Mitigation strategies that pinpoint the root cause of HS-induced intestinal permeability are vital as they can provide an efficient solution to the said matter. Nutritional interventions are proven effective in alleviating HS’s effects on the intestinal integrity of pigs [18]. As HS induces cellular OS in the intestine [5], this review aims to look into the details on how HS and its stressors promote intestinal permeability and the ability of antioxidants to mitigate such adverse effects.

## 2. Intestinal Epithelium

A healthy gut is important in aiding efficient digestion and absorption of the dietary nutrients ingested by an animal [19]. The intestine’s digestive, absorptive and protective functions are dependent on an intact and functional intestinal epithelium (IE) [20]. As an active barrier, it serves both the uptake of nutrients and prevents harmful substances and potential pathogens from entering into the bloodstream [21,22]. The intestine is inhabited by many microorganisms, which provides an avenue for nutrition, metabolism and immunity [23]. A single layer of intestinal epithelial cells (IECs) bound together by TJs (composed of transmembrane proteins: occludin, claudins and junctional adhesion molecules) makes up the IE [24,25]. This unique composition of the IE is to prevent harmful microorganisms, antigens and toxins from the gut lumen from entering into the circulation [26]. This is achieved through the IEC’s function in creating mucosal barrier (physical and chemical barrier) which maintains symbiosis between the gut microbiota and the host. The barriers maintain homeostasis by segregating gut microbiota and host immune cells, which prevents inflammation due to excessive immune response [27]. IECs also influence the recruitment and activation of immune cells through production of cytokines and chemokines [22,28], thus being appreciated as a central component of innate immunity [29]. As a single cell layer, IE is selectively permeable through transcellular (nutrients passing through the cell) and paracellular (via TJ) pathways [30]. The IEC maintains barrier integrity through weak protein–protein bonding of junctional complexes such as TJ, adherens junction (AJ) and desmosomes, all of which have occlusive properties [31]. Proteins that link adjacent epithelial cells to the actin cytoskeleton are present in the AJ and desmosomes which are essential for mechanical linking of cells [9,32]. The TJ regulates the formation of intestinal barriers by modulating cell proliferation, differentiation and polarization [33]. It is also responsible for the regulation of ions, solutes and water across the intestinal epithelium through paracellular movement [34]. The pigs’ IE renews every two to three days, compelled by the intestinal stem cells (ISCs) [35]. This ensures that only the fittest and metabolically able cells comprise the IE and maintain an impermeable barrier to gut microbiota and luminal contents as well as for nutrient digestion, absorption and secretion of antimicrobial peptides [36]. Another positive influence of ISCs is the generation of highly proliferative transit-amplifying cells. These cells then differentiate into enterocytes and secretory cells upon migration to the villi and into Paneth cells (secrete antimicrobial peptides) upon migration towards the crypt. Although Paneth cells’ existence in the pig’s intestine is debatable [37], several researches have confirmed that it exists [38,39]. Stability of ISC’s self-renewal and differentiation controls the intestinal epithelial homeostasis and is essential for ensuring intestinal epithelial integrity [35].

## 3. Heat Stress and Its Induced Stressors

HS is experienced by pigs when they can no longer release excess heat as influenced by increase in environmental temperature. The sensitivity of pigs to HS is due to their thick layer of subcutaneous adipose tissue and lack of functional sweat glands, making them less efficient in losing heat when exposed to high environmental temperature [40]. However, ambient temperature (AT) is not the sole culprit in inducing HS to pigs, as studies show that it is also influenced by relative humidity (RH) and the index that combines these factors is called heat index (HI). Knowledge about HI is important as it can alert farmers to provide appropriate response to either prevent or alleviate the consequences of HS on the performance of pigs. HS index charts released by Iowa State University and Ontario Ministry of Agriculture show that pigs are in danger of experiencing HS at AT of 26 °C with an RH of 75 to 90% [41,42]. This indicates that even at temperatures within the desirable limits for pigs, it still makes them susceptible to HS when the RH is high. This can be supported by the fact that, under such temperatures, pigs tend to loss more heat through evaporation (panting); however, it is insufficient as higher RH makes it less effective due to the fact that less moisture can be evaporated. However, when the AT is at 30 °C and above, RH under 50% is enough to cause HS to pigs [42,43]. Several research pieces suggest that HS induces OS in various species of food animals such as poultry [44] and pigs [5,45]. HS is considered cytotoxic and has been proven to induce oxidative damage by disturbing mitochondrial homeostasis and cellular function [4]. Intestinal homeostasis is lost due to OS and associated tissue redox imbalance, which negatively affects the intestine’s digestive and absorptive function and its purpose for stem cell proliferation and immune response [46]. The latter is confirmed by cellular apoptosis inhibition caused by HS [3]. HS induces hypoxia in the intestine, resulting in the imbalance between the production of ROS and the antioxidant defense system, which jeopardizes the intestinal epithelium and enhances inflammatory response which can exacerbate intestinal permeability [10,47]. Such response is mediated by various immune cells (macrophages, dendritic cells, mast cells and lymphocytes) and their mechanisms in antigen processing and presentation and production of regulatory and effector molecules (cytokines, chemokines and immunoglobulins). T lymphocytes identified by a surface cluster of differentiation (CD) molecule named CD3 has two major groups, CD4 (helper T cells) and CD8 (cytotoxic T cells), which are found in the intestinal epithelium and can contribute to the barrier integrity but occasionally become proinflammatory when the balance between the groups is disturbed [48,49]. In the study of Huo et al. [3], pigs subjected to chronic HS expressed significant increase of CD3, but were associated with an imbalance between CD4 and CD8 signifying immune dysfunction. Nevertheless, in other studies inflammatory response to HS involved upregulation of proinflammatory cytokines (tumor necrosis factor alpha (TNFα), interleukin (IL-4 and IL-6) in the skeletal muscle of pigs and in the intestine of cattle and chicken [4,50,51]. Hypoxia-inducible factor (HIF)-1 triggers the cellular response to hypoxia. HIF-1 consists of two subunits: the oxygen-regulated alpha (α) and a constitutively expressed beta (β). The former is responsible for the regulation of energy homeostasis and cellular adaptation to hypoxia. Under normal conditions, HIF-1α is rapidly degraded in the proteasome through hydroxylation by oxygen-sensitive prolyl hydroxylases (PHD). However, hypoxic conditions can lead to HIF-1α’s rapid accumulation, stabilization and transcriptional activity due to the inhibition of PHD activity, which enables the cells to adapt to hypoxic stress. Nevertheless, the consequence of hypoxic signaling is the abnormal accumulation of ROS by cytochrome reductase of the mitochondrial electron transport chain [47,52]. ROS are unstable and oxygen-centered molecules containing unpaired-valence electrons which are highly reactive with proteins, lipids, carbohydrates and nucleic acids in the cell. It includes radical compounds (superoxide (O_2_^−^), hydroxyl radicals (HO), lipid hydroperoxides) and reactive non-radical compounds or oxidants (oxygen (^1^O_2_), ozone (O_3_), hydrogen peroxide (H_2_O_2_), hypochlorous acid (HOCl), hypobromous acid (HOBr), chloramines (RNHCl) and organic hydroperoxides (ROOH)), which can impair intestinal cells, leading to OS [53,54,55]. The animal body is equipped with antioxidant defense system which is responsible against the deleterious effect of ROS. This system involves endogenous enzymatic antioxidants (superoxide dismutase (SOD), catalase (CAT), glutathione peroxidase (GPx) and glutathione reductase (GR)) and non-enzymatic antioxidants (glutathione (GSH), thioredoxin (Trx) and melatonin (Mel)) with ability to remove free radicals from the system and inhibit oxidation. Among the various endogenous antioxidant enzymes, SOD and CAT impart substantial antioxidant defenses against ROS [55]. SOD catalyzes dismutation of O_2_^−^ into O_2_ and H_2_O_2_, it scavenges O_2_^−^ from mitochondrial intermembranous space and mitochondrial matrix, while CAT dismutates H_2_O_2_ to H_2_O and O_2_ [56]. Other antioxidant enzymes also have a significant role; GPx is responsible for the conversion of GSH into glutathione disulfide (GSSG), which reduces H_2_O_2_ to H_2_O and lipid hydroperoxides to stable alcohols. Conversely, GR can reduce GSSG to GSH which maintains the balance of cell differentiation, growth and apoptosis [53,57,58]. Non-enzymatic antioxidants such as GSH, Trx and Mel interrupt free radical chain reaction. GSH is ubiquitously expressed together with GSH-Px, GR and glutathione *S*-transferases (GST) forming the glutathione system which serves as an antioxidant barrier in the gut mucosa. Trx serves as donors of electron to peroxidase to efficiently scavenge ROS, while Mel can efficiently protect the mitochondria from oxidative damage through its direct free radical scavenging capability; whilst other antioxidants can be converted into free radicals, the said conversion does not apply to Mel as its oxidative role involves donation of two electrons. These mechanisms can then result in reduced lipid peroxidation, DNA damage, protein activation and restoration of intestinal injury induced by OS [53,55,59]. Antioxidant to pro-oxidant balance in the intestine is an essential determinant of human [60] and pig gut health [61]. However, this balance is disturbed by HS, as it increases mitochondrial free radicals such as O_2_^−^ and oxidants (H_2_O_2_), and decreases the activities of endogenous antioxidants which are associated with the excessive generation of ROS [7,62]. Normal cellular metabolism produces low to moderate amounts of ROS as its byproducts, which is useful in several physiological processes such as wound healing, tissue repairs as well as killing invading pathogens [55]. However, its overproduction due to HS can be detrimental as heat-induced ROS targets proteins; lipids, polysaccharide and DNA [7]. ROS directly reacts with DNA components which can cause DNA lesions, particularly the mitochondrial DNA (MtDNA) as its location is close to the ROS-generated place. This then leads to mutations that can cause the electron transport chain (ETC) complex dysfunction and lead to more production of ROS and oxidative damage [7,63,64]. Oxidation of proteins can be induced by ROS, as it oxidizes various amino acids causing formation of protein-to-protein cross linkages that can result in protein denaturation and loss of functioning as well as losses in enzyme activity, function receptors and transport proteins [63,65]. Lipids are highly susceptible to oxidation by free radicals such as HO; in excess it can damage cell membrane and lipoproteins through a process called lipid peroxidation and leads to the formation of malondialdehyde (MDA) and production of 4-hydroxenonenal (4-HNE) which are cytotoxic and mutagenic. As this process occurs through radical chain reaction, it spreads rapidly and affects numerous lipid molecules [66]. Thus, excessive ROS can lead to cellular dysfunction, disruption of vital cellular processes and apoptosis through modification of the structure of cellular proteins and the alteration of their function [7,67,68,69].

## 4. Intestinal Integrity and Function of Pigs under Heat Stress

Harmonized regulation of the mucus layer, TJs, IECs and the enteric immune system influences the intestine’s integrity and function [9,32]. The first line of defense against intestinal injury is provided by the intestinal mucus layer [70], as it serves as a physical barrier against bacteria and antigenic substances in the lumen by coating the interior surface of the intestine and lubricating its luminal contents [71]. It is composed of mucin glycoprotein (MUC2) and bioactive molecules such as epithelial-bound mucins (MUC1, MUC3 and MUC17), which are synthesized by the intestinal goblet cells [72] that are confined to the crypts of Lieberkühn and on the small intestinal villi. In the colon, goblet cells amass at the opening of the colonic crypts and are also found deep within the crypts and on the surface of the colon [73,74]. The high polymeric protein backbone structure of these mucins that are linked to numerous hygroscopic and hydrophilic oligosaccharide side-chains contributes to the mucus layers’ gel-like structure [72,75]. The intestinal mucus layer establishes an active semi-permeable barrier which allows passage of nutrients from the gut lumen towards the epithelium [76] while promoting clearance that separates bacteria from the epithelial cells that inhibit inflammation and infection [77]. HS can directly and indirectly (via reduced feed intake) compromise intestinal integrity of pigs. The former takes effect as HS induces hypoxia and OS in the intestine, as assessed by acute increase of HIF-1α mRNA abundance and increase in lipid oxidation (4-HNE abundance). Moreover, intestinal structures of proteins responsible for cell structure and motility (such as alpha-actinin-1 and myosin regulatory light chain) as well as for cellular proliferation and apoptosis (TNF receptor-associated protein 1 and Erlin-2) are altered by HS along with reduction of endogenous antioxidants (GPx and GSH). This leads to deprivation of oxygen and nutrients to the enterocytes and loosening of TJs. Nutrient restriction due to reduced feed intake also causes alterations in intestinal function and morphology of pigs, thus accompanied by HS’s direct effects exacerbating intestinal permeability [5,17,78,79] with an emphasis on intestinal morphology parameters (Table 1), such as reduced villus height and crypt depth, villus to crypt ratio and decrease in the mucosal surface, sloughing of the intestinal villi as well as undergoing autolysis, regardless of the duration of exposure [17,80,81,82]. This then influences changes in the cellular proliferation and membrane function [83]. Intact morphological structure of the intestine is important for nutrient utilization and absorption and it is associated with longer villi which is a good indicator of a healthy gut; thus, its reduction and damage can be cautiously used to represent increasing intestinal permeability and infiltration of endotoxins [81,84].

Nevertheless, these consequences on intestinal integrity can be influenced by the duration and intensity of the pigs’ exposure to HS. During the first 2 to 4 h of HS (37 °C and 40% RH), the pigs’ intestinal integrity in the ileum declined, associated with decrease in transepithelial resistance (TER), while the colon remained unaffected at this period. Between 6 and 12 h TER rebounds as it increases, however it ultimately declined after 24 h of exposure signifying intestinal permeability [80,86,87]. Under such conditions, Pearce et al. [80] observed increase in ileum MUC2 after 6 h of exposure to HS which can act as a protective barrier for the intestine and may combat the decrease in intestinal integrity. However, in another study, pigs exposed to HS for 3 h showed a reduction of goblet cells in the jejunum and ileum [85], and this was also observed even during the recovery period of 7 days after constant exposure to HS for 3 days [81]. This suggests that mucin glycoprotein (MUC2) production and activity can be reduced as this is produced by the goblet cells which can then potentially compromise intestinal function; that could lead to higher risk of infection caused by increased bacterial adhesion to the epithelium [85,88]. Pearce et al. [16] observed decreased resistance across the intestinal epithelium of pigs under HS, as assessed by reduced TER, which favors increasing intestinal permeability and allows the translocation of lipopolysaccharides (LPS) into the systemic circulation; that leads to endotoxemia and increases inflammation [89,90].

**Figure 1:**
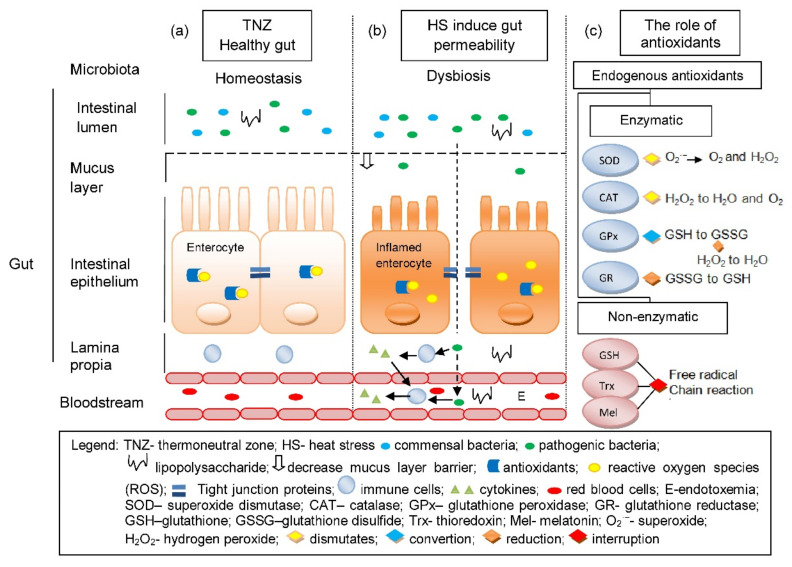
The condition of the gut under thermoneutral zone (**a**) is normal and is considered a healthy gut, as there is a homeostasis in microbiota, antioxidant to ROS balance and a fully functional gut epithelium that prevents the entry of bacteria and toxins. (**b**) HS induces gut permeability, which causes OS due to the imbalance between the endogenous antioxidants and ROS. It also allows translocation of pathogenic bacteria and toxins into the blood circulation via paracellular transport due to the disruptions of TJs and can lead to endotoxemia. (**c**) The role of endogenous antioxidants in cellular redox balance through elimination of ROS.

TJ, which is responsible for the intestine’s physical barrier integrity, can be compromised under HS with evidence of its altered expression and localization. Pigs under 24 h of exposure to HS showed increased claudin 3 and occludin in the ileum [16]. Claudin 3, acts as a sealing component of TJ and occludin is essential for TJ integrity [91] Increase in the TJ proteins may indicate improvement of the intestinal barrier during HS as it strives to relieve the stress-induced permeability; this is often associated with heat-induced expression of heat shock proteins (HSPs) [89]. Recovery of the intestine from HS is influenced by HSPs (HSP27, 70 and 90). These molecular chaperones aid in protein folding and cell survival under stress conditions. Expression of these proteins was upregulated in the ileum and colon of pigs within 2 to 4 h of exposure to HS; such upregulation might influence the partial recovery from HS. Liu et al. [92] observed severe damage in the small intestine (epithelium shedding at the tips of the intestinal villi, and shorter villus height and shallower crypt depth for the duodenum and jejunum) of Chinese mini pigs exposed to HS (40 °C) 5 h a day for 3 days. However, they also observed gradual recovery from the pigs exposed to the same condition on the 6th day of exposure until day 10; still, it is incomparable to those pigs in the thermoneutral group. This gradual recovery is possibly influenced by HSPs ability to prevent the activation of conventional protein kinase C (cPKC), resulting in reduced myosin light chain kinase (MLCK) protein phosphorylation of the actin cytoskeleton [80,92]. Noticeably, TJ proteins distribution was altered; as Pearce et al. [16] observed, upregulation of claudin protein was more in the membrane fraction, while occludin was more in the cytosolic fraction. It can be said that the regulation of TJ complexes is disturbed with emphasis on key kinases, particularly in a c-Srcfamily kinases (SFKs) manner [16,93]. Multiple kinases influence on occludin phosphorylation is believed to contribute to the regulation and modification of TJ; involvement of SFKs in the assembly and integrity of TJ are evident by regulating cell proliferation, migration, differentiation and adhesion [94,95]. Disassociation of TJ protein complexes was observed upon activation and upregulation of Casein kinase II-α (CKII-α) in the ileum of pigs under HS which impaired its barrier function [16]. Indeed, as observed in mice, CKII-α plays significant roles: in occludin phosphorylation and as regulator of zonula occluden-1 (ZO-1), claudin-1 and claudin-2 proteins [96]. Moreover, increased expression and activation of MLCK were observed and associated with reduced intestinal integrity [16]. MLCK largely mediates the actin cytoskeleton regulation in the epithelial cells, which affects its role in TJ physiology and intestinal integrity [97]. In a different study, where finishing pigs were subjected to cyclical HS (35 °C for 12 h and 22 °C for 12 h) for 30 days, reduced expression TJ proteins ZO-1 and occludin were observed compromising their epithelial barrier function [98]. Despite the varied intensity of HS, intestinal integrity and function of pigs is compromised as assessed by HS’s effects on the intestinal mucus layer, TJs, enteric immune and antioxidant system which are condensed in Table 2. The condition of the gut under thermoneutral zone (TNZ) and HS is illustrated in Figure 1 along with the role of endogenous antioxidants in suppressing ROS.

## 5. Mitigation of Heat-Stress-Induced Intestinal Permeability by Antioxidants

Nutrition is one of the promising avenues to aid HS and its induced stressors, particularly to the intestinal integrity and function of pigs [99]. Antioxidants such as vitamins and minerals are a potential nutritional tool to compensate for HS’s harmful effects [100]. These substances can reduce cell damage by neutralizing free radicals [101] during HS-induced oxidation and oxidative stress [7]. Nevertheless, the body is capable of synthesizing antioxidant enzymes, including GPx, SOD and CAT, and non-enzymatic antioxidants (GSH, Trx, melatonin and coenzyme Q); exogenous antioxidants (vitamins A, C, D, E and minerals selenium, iron, copper, zinc and manganese) which are obtained through the diet are still essential as they serve as co-factors of the said antioxidant enzymes for optimum catalytic activity [102]. In vitro studies proved that gastrointestinal cell homeostasis is influenced by vitamins A and D as these substances can modify the expression of TJ molecules and upregulate ZO-1, occludin and other claudins that lead to increased transepithelial resistance (TER) of the intestinal epithelial cells in mice and regulate its barrier function [48,103]. He et al. [104] reported that vitamin A alone can enhance the TER by alleviating LPS-induced intestinal permeability. Vitamin D with known biological functions such as vitamin D receptor (VDR) can maintain the mucosal barrier integrity by reducing MLCK [105]. Vitamin C’s antioxidant function is to protect cellular structures from the harmful effect of free radicals. In contrast, as a lipid-soluble antioxidant, vitamin E can neutralize free radicals induced by HS [106]. Besides its antioxidant function, vitamin C is also responsible for the synthesis of serotonin, which is involved in the proper functioning of the endocrine, nervous, digestive and immune systems [107,108]. Minerals such as Selenium (Se) and Zinc (Zn) have their diverse antioxidant function. Se incorporates into proteins in the form of selenoproteins (antioxidant enzymes) to avert cell damage and OS [109]. Zn delays the oxidative process by inducing metallothionein’s expression (responsible for zinc-related cell homeostasis) and behaves as potent electrophilic scavengers and cytoprotective agents [110]. Furthermore, the antibacterial effects of Zn have beneficial effects on the gut health of pigs [111]. Several research pieces also proved that independent and combined use of these minerals as supplements improved the condition and performance of rabbits, lambs and broilers exposed to high environmental temperature [112,113,114].

With these notable facts about antioxidants, its potential has been explored as a sole supplement and in combination as a means to alleviate HS’s ill effects on the intestinal integrity and function of pigs (Table 3).

Sanz Fernandez et al. [115] reported that supplementation of 200 mg organic Zn (zinc amino acid complex, ZnAA) could improve the intestinal integrity of pigs under severe HS. Regulated dietary organic zinc (ZnAA 60 mg/kg) with the addition of inorganic Zn (zinc sulfate 60 mg/kg) supplementation improved the intestinal barrier integrity as well as reduced blood endotoxin of pigs under acute heat stress (12 h) by exhibiting higher ileum TER as compared to pigs in the HS condition without ZnAA supplementation. Furthermore, villi height to crypt depth ratio of HS pigs supplemented with ZnAA is comparable to pigs under thermal comfort and with no manifestation of ileal autolysis, unlike the pigs in the HS group without ZnAA supplementation [87]. Liu et al. [116] reported that combinations of Zn oxide and tannins (ZnO + hydrolysable tannins) improved the antioxidant capacity and digestive enzymes of pigs. Organic zinc (Zn glycinate and Zn methionine) also influences the pigs’ antioxidant status and helps maintain a healthy immune system [117,118]. In another study, supplementation of Se and vitamin E at high levels attenuated HS’s impact on the intestinal barrier integrity of pigs, associated with a depletion of OS, as assessed by decrease in antioxidant enzymes [119]. This was in agreement with the previous report of Lv et al. [120], which shows the capability of Se to enhance antioxidant capacity as evidence of increased blood GPx activity and a higher concentration of thyroid hormones that promotes a stable and healthy gastrointestinal ecosystem of pigs under HS. 

Recently, a study by Liu et al. [15] revealed that supplementing pigs with dietary selenium-enriched yeast attenuated OS-induced disruption of intestinal mucosa, with elevated mucosal CAT, GPx and total antioxidant capacity (T-AOC) activities and improved intestinal barrier functions with enhanced TJ protein (ZO-1) distribution and abundance as well as enhanced intestinal morphology. Summary of antioxidants’ mitigation on HS’s adverse effects on intestinal integrity and morphology of pigs is shown in Table 4.

The supplementation of antioxidants influence on the intestinal integrity and function of pigs under HS positively impacts their performance. The capability of antioxidants to protect the intestinal barrier integrity and increase the levels of antioxidant enzymes can lead to better gut health, intestinal function, alleviation of endotoxemia and OS. Such abatement on the intestinal integrity components signifies better nutrient digestion and absorption which can be summed up as a better production performance of pigs under HS, as supported by the pigs’ manifestation of better growth, intestinal barrier function, enhanced immune function and improved antioxidant system [115,118,119].

## 6. Conclusions

HS induces intestinal OS in pigs, compromising intestinal homeostasis and jeopardizing the intestinal epithelium. Based on the information from various researches, as HS progresses, duodenal, jejunal and ileal villus height, crypt depth and villus height to crypt depth ratio are reduced by about 11%, 3% and 9%, respectively. This indicates that HS causes intestinal damage mainly in the duodenum and ileum. HS also threatens the intestine’s protective function against harmful substances as it reduces the expression of TJ proteins and the intestine’s TER. This leads to the disruption of the TJ barrier function, allowing translocation of endotoxins into the systemic circulation and leading to endotoxemia. Dietary antioxidants (zinc, vitamin E and selenium) can combat HS-induced OS by neutralizing free radicals and serving as co-factors for antioxidant enzymes. In conclusion, the antioxidant supplementation can partially mitigate the effect of heat stress on the villus height and TER by about 70–85%. Interestingly, the average mitigation of crypt depth shows negative result, but the individual results are variable. We have to point out that only a few studies applied the necessary treatments to allow the calculation of % mitigation, which indicates the necessity of further research. We also have to admit that the dietary interventions in the reviewed manuscripts mainly lasted only for the length of the study. Therefore, it would be worth studying whether the full intestinal recovery can be achieved after HS with longer specific supplementation. Due to the complex nature of the problem, combined approaches may have higher mitigation capacity.

## Figures and Tables

**Table 1:** Intestinal morphology of pigs under thermal comfort (TC) and heat stress (HS).

Parameters	Type of HS ^a^	TC (20–22 °C) RH ^c^ (50–65%)	HS (30–40 °C) RH (35–60%)	% change ^b^	Average Body Weight, kg	Reference
Villus height, µm						
Duodenum	Cy ^d^	327	317	−3.05	7.6	[84]
Jejunum	Cy ^d^	333	317	−4.8	7.6	[84]
	Cy ^e^	579	509	−12.1	19.5	[81]
	Cy ^f^	603	506	−16.1	6.8	[82]
	Cy ^g^	443	333	−24.8	78.5	[85]
	Ch ^h^	533	455	−14.6	79.0	[5]
	Ch ^i^	499	385	−22.9	48.8	[17]
Average		498.3	417.5	−16.2		
Ileum	Cy ^d^	309	292	−5.5	7.6	[84]
	Cy ^e^	364	370	+1.7	19.5	[81]
	Cy ^g^	379	280	−26.1	78.5	[85]
Average		350.7	314.0	−10.4		
Intestinal average		392.0	349.7	−10.8		
Crypt depth, μm						
Duodenum	Cy ^d^	212	209	−1.4	7.6	[84]
Jejunum	Cy ^d^	210	196	−6.7	7.6	[84]
	Cy ^e^	466	412	−11.6	19.5	[81]
	Cy ^f^	163	164	+0.6	6.8	[82]
	Cy ^g^	301	294	−2.3	78.5	[85]
	Ch ^h^	182	162	−11.0	79.0	[5]
	Ch ^i^	289	276	−4.5	48.8	[17]
Average		268.5	250.6	−6.7		
Ileum	Cy ^d^	201	203	+1.0	7.6	[84]
	Cy ^e^	350	357	+2.0	19.5	[81]
	Cy ^g^	221	218	−1.4	78.5	[85]
Average		257.3	259.3	+0.8		
Intestinal average		245.9	239.6	−2.5		
Villus: crypt depth						
Duodenum	Cy ^d^	1.54	1.52	−1.3	7.6	[84]
Jejunum	Cy ^e^	1.31	1.29	−1.5	19.5	[81]
	Cy ^g^	1.54	1.17	−24.0	78.5	[85]
	Ch ^h^	2.95	2.83	−4.1	79.0	[5]
	Ch ^i^	1.70	1.40	−17.7	48.8	[17]
Average		1.88	1.67	−11.2		
Ileum	Cy ^d^	1.53	1.43	−6.5	7.6	[84]
	Cy ^e^	1.15	1.14	−0.9	19.5	[81]
	Cy ^g^	1.74	1.30	−25.3	78.5	[85]
Average		1.47	1.29	−12.2		
Intestinal average		1.63	1.49	−8.6		

^a^ Type of HS: Cy—Cyclic, Ch—Chronic; ^b^ % change: (TC-HS)/(TC) × 100; ^c^ RH—relative humidity; ^d^ five hours exposure to 40 °C each day for 10 days (minipigs two month old); ^e^ three days exposure to 33.6 °C; ^f^ five hours exposure to 40 °C each day for 9 days; ^g^ three hours exposure to HS (38.51 °C); ^h^ under HS (30 °C) for 21 days; ^i^ seven days exposure to HS (35 °C).

**Table 2:** Heat stress (HS) effects on the intestinal integrity and function of pigs.

Parameter	HS Effects	HS Intensity	RH a, %	HS Length	References
Intestinal mucus layer	Reduction of goblet cells in jejunum and ileum which leads to the decreased production of mucin	33.6 °C, 35 °C, 38.51 °C	30–40	3, 12 h and 3 days	[81,85,98] ^b,c,d^
Tight junction	Altered expression and localization and reduced expression of TJ proteins (ZO-1 and occludin), TER reduction in the jejunum and ileum and manifestation of endotoxemia	35 °C, 38 °C	35–43	12, 24 h and 7 days	[16,17,98] ^e,f,d^
Enteric immune system	Disruptions were observed through inhibition of cellular apoptosis and gut permeability	35 °C, 39 °C	43	24 h and 10 days	[3,86] ^g,h^
Antioxidant system	Decreased glutathione concentration and imbalance of ROS and endogenous antioxidants in the jejunum and ileum	30 °C, 35 °C	35–60	7 and 21 days	[5,17] ^i,f^

^a^ RH—relative humidity; ^b^ (average live weight 19.5 kg); ^c^ (average live weight 79.0 kg); ^d^ (average live weight 70.2 kg); ^e^ (average live weight 46.0 kg); ^f^ (average live weight 48.0 kg); ^g^ (85–95 days old miniature pigs); ^h^ (average live weight 46.0 kg); ^i^ (average live weight 79.0 kg).

**Table 3:** Effects of antioxidants on the intestinal integrity and function of pigs under heat stress.

Parameter	Antioxidants	Con ^a^ mg/kg	Supp ^b^ mg/kg	Effects	References
Intestinal barrier integrity	SeY ^c^ ZnAA + ZnSO_4_^d^ ZnAA + ZnSO_4_^d^	0 0 + 120 0 + 120	250 200 + 120 60 + 60	Improved intestinal TJ, high ileum TER, reduction of blood endotoxin and improved intestinal histology and morphology	[15] ^g^ [87] ^h^ [115] ^i^
Antioxidant system	SeY ^c^ Se and VE ^e^ SeP ^f^	0 0.5 and 100 0.16	250 1 and 200 0.46	Elevation of antioxidant enzymes (catalase and glutathione peroxidase) and enhanced mucosal antioxidant capacity	[15] ^g^ [119] ^j^ [120] ^k^

^a^ Con—levels in the control diet; ^b^ Supp—supplemented in the experimental diet; ^c^ SeY—selenium-enriched yeast; ^d^ ZnAA + ZnSO_4_—zinc amino acid complex and zinc sulfate; ^e^ Se—selenium and VE—vitamin E; ^f^ SeP—selenium enriched probiotic; ^g^ (average live weight 7.30 kg); ^h^ (average live weight 64.0 kg); ^i^ (average live weight 43.0 kg); ^j^ (average live weight 20.0 kg); ^k^ (average live weight 7.9 kg).

**Table 4:** Mitigation of heat stress effect on intestinal morphology and TER by antioxidant supplementation.

Heat and Dietary Treatment	TC ^a^ (20–22 °C) RH ^d^ (35–60%)	HS ^b^ (30–40 °C) RH (40–50%)	>HAS ^c^ (30–40 °C) RH (40–50%)	
Antioxidants	Supplementation mg/kg (in the diet)			Reference
Zn ^e^	25	25	2525	[121] ^h^
vitamin E	18.1	18.1	200	[122] ^i^
ZnAA ^f^ + ZnSO_4_	0 + 120	0 + 120	200 + 120	[87] ^j^
ZnAA ^f^ + ZnSO_4_	0 + 120	0 + 120	60 + 60	[115] ^k^
Se + vitamin E	0.5 + 100	0.5 + 100	1 + 200	[119] ^l^
Without Antioxidants	Basal diet		-	[5,17,81,82,84,85] ^m,n,o,p,q,r^
Parameters				% mitigation ^s^
Villus height, μm				
Duodenum	na ^t^	528 ^h^	550	
	327	317 ^q^	na	
Jejunum	na	459 ^h^	508	
	425	368 ^i^	378	+17.5
	533	455 ^m^	na	
	499	385 ^n^	na	
	579	509 ^o^	na	
	603	506 ^p^	na	
	333	317 ^q^	na	
	443	333 ^r^	na	
Ileum	na	391 ^h^	408	
	na	310 ^i^	332	
	428	342 ^j^	393	+59.3
	364	370 ^o^	na	
	309	292 ^q^	na	
	379	280 ^r^	na	
Intestinal average	435	385	428	+86
Crypt depth, μm				
Duodenum	na	341 ^h^	333	
	212	209 ^q^	na	
Jejunum	na	243 ^h^	265	
	148	130 ^i^	106	−133.3
	182	162 ^m^	na	
	289	276 ^n^	na	
	466	412 ^o^	na	
	163	164 ^p^	na	
	210	196 ^q^	na	
	301	294 ^r^	na	
Ileum	na	235 ^j^	254	
	na	134 ^i^	114	
	357	201 ^j^	262	+39.1
	350	357 ^o^	na	
	201	203 ^q^	na	
	221	218 ^r^	na	
Intestinal average	258.3	236.0	222.3	−61
Villus: crypt depth				
Duodenum	na	1.56 ^h^	1.67	
	1.54	1.52 ^q^	na	
Jejunum	na	1.91 ^h^	1.92	
	2.92	2.90 ^i^	3.66	+3800
	2.95	2.83 ^m^	na	
	1.70	1.40 ^n^	na	
	1.31	1.29 ^o^	na	
	1.54	1.17 ^r^	na	
Ileum	na	1.69 ^h^	1.62	
	2.53	2.49 ^i^	3.06	+1425
	1.20	1.70 ^j^	1.50	+40.0
	1.15	1.14 ^o^	na	
	1.53	1.43 ^q^	na	
	1.74	1.30 ^r^	na	
Intestinal average	1.82	1.62	2.24	+310
TER ^g^, Ω/cm^2^	200	140 ^j^	183	+71.7
	115	82 ^k^	105	+69.7
	60.4	46.7 ^l^	56.7	+73.0
Average	125	89.6	115	+72

^a^ TC—thermoneutral zone and control feed; ^b^ HS—heat stress and control feed; ^c^ HSA—heat stress and antioxidant fortified feed; ^d^ RH—relative humidity; ^e^ Zn—as zinc oxide; ^f^ ZnAA + ZnSO_4_—zinc amino acid complex + zinc sulfate; ^g^ TER—transepithelial resistance; ^h^ average live weight 10.45 kg; ^i^ average live weight 6.62 kg; ^j^ average live weight 64.0 kg; ^k^ average live weight 43.0 kg; ^l^ average live weight 20.0 kg; ^m^ average live weight 79.0 kg; ^n^ average live weight 48.8 kg; ^o^ average live weight 19.5 kg; ^p^ average live weight 6.8 kg; ^q^ minipigs two month old average live weight 7.6 kg; ^r^ average live weight 78.5 kg; ^s^ % mitigation: (HSA-HS)/(TC-HS) × 100; ^t^ na—not available.

## Data Availability

No new data were created or analyzed in this study. Data sharing is not applicable to this article.
